# Heterologous fibrin biopolymer as an emerging approach to peripheral nerve repair: a scoping review

**DOI:** 10.1590/1678-9199-JVATITD-2023-0060

**Published:** 2024-04-15

**Authors:** Kevin Silva Muller, Felipe Cantore Tibúrcio, Rui Seabra Ferreira, Benedito Barraviera, Selma Maria Michelin Matheus

**Affiliations:** 1 Department of Structural and Functional Biology, São Paulo State University (UNESP), Botucatu Institute of Biosciences, Botucatu, SP, Brazil.; 2 Botucatu Medical School, São Paulo State University (UNESP), Botucatu, SP, Brazil.; 3 Center for the Study of Venoms and Venomous Animals (CEVAP), São Paulo State University (UNESP), Botucatu, SP, Brazil.; 4 Center for Translational Sciences and Biopharmaceuticals Development (CTS), Center for the Study of Venoms and Venomous Animals (CEVAP), Botucatu, SP, Brazil.

**Keywords:** Fibrin sealant, Neurorrhaphy, Nervous system, Regenerative medicine

## Abstract

Nerve injuries present a substantial challenge within the medical domain due to their prevalent occurrence and significant impact. In nerve injuries, a range of physiopathological and metabolic responses come into play to stabilize and repair the resulting damage. A critical concern arises from the disruption of connections at neuromuscular junctions, leading to profound degeneration and substantial loss of muscle function, thereby hampering motor tasks. While end-to-end neurorrhaphy serves as the established technique for treating peripheral nerve injuries, achieving comprehensive morphofunctional recovery remains a formidable challenge. In pursuit of enhancing the repair process, alternative and supportive methods are being explored. A promising candidate is the utilization of heterologous fibrin biopolymer, a sealant devoid of human blood components. Notably, this biopolymer has showcased its prowess in establishing a stable and protective microenvironment at the site of use in multiple scenarios of regenerative medicine. Hence, this scoping review is directed towards assessing the effects of associating heterologous fibrin biopolymer with neurorrhaphy to treat nerve injuries, drawing upon findings from prior studies disseminated through PubMed/MEDLINE, Scopus, and Web of Science databases. Further discourse delves into the intricacies of the biology of neuromuscular junctions, nerve injury pathophysiology, and the broader utilization of fibrin sealants in conjunction with sutures for nerve reconstruction procedures. The association of the heterologous fibrin biopolymer with neurorrhaphy emerges as a potential avenue for surmounting the limitations associated with traditional sealants while also mitigating degeneration in nerves, muscles, and NMJs post-injury, thereby fostering a more conducive environment for subsequent regeneration. Indeed, queries arise regarding the long-term regenerative potential of this approach and its applicability in reconstructive surgeries for human nerve injuries.

## Background

Peripheral nerves are some of the most delicate structures in the human body, prone to easily being damaged by injuries such as compressions, crushes, and traumas [1]. Nerve injuries are a common clinical case with a high global incidence, affecting around 18 individuals per 100,000 each year [2]. Many individuals affected by nerve injuries, both in severe and moderate cases, have incomplete recovery, resulting in transient or permanent losses of motor and sensory functions, as well as chronic pain, muscle atrophy, and weakness. Approximately one-third of all nerve injuries demonstrate incomplete recovery with poor restoration of function [3]. Several factors hinder axonal regeneration, including severity, mechanism of injury, the large distance between the neuron cell body and target tissue, and the loss of regenerative support from Schwann cells (nerve glial cells) after injury, so that effective functional recovery is typically not achieved [4, 5]. The proper reinnervation of neuromuscular junctions (NMJs) by the nerve terminal plays an important role in obtaining a functional NMJ and, thus, a positive outcome.

Upon transection/neurotmesis, all structures of the neuromuscular apparatus are hindered, from the muscle fibers up to the motor neuron cell body in the spinal cord, undermining the quality of life of patients. Despite advances in microsurgical repair techniques, researchers have not yet achieved satisfactory functional recovery of the affected structures. Even with successful reconnection between the nerve stumps, the rates of motor recovery only reach a 50% success rate [6]. Then, the development of new strategies and approaches aimed at better regeneration is necessary. In this way, a new heterologous fibrin biopolymer (HFB) calls out attention as a promising candidate for adjunct therapy, as it has been showing positive results in a range of regenerative treatments, such as bone, tendon, spinal cord, and skin injuries, developing a more permissive environment for regeneration.

The current HFB consists of two major components, namely a thrombin like-enzyme (serine protease) extracted from the *Crotalus durissus terrificus* venom and a cryoprecipitate rich in fibrinogen, obtained from the blood of the buffalo *Bubalus bubalis* [7]. When both components are mixed with the calcium chloride diluent, the product polymerizes, forming a robust fibrin network. HFB has an activity comparable to commercial sealants, as it is biodegradable, bioabsorbable, and has no toxicity or adverse reaction [8]. Because of its unique composition and absence of human blood, it is considered the only heterologous fibrin biopolymer in the world [9].

The use of HFB as an adjunct in peripheral and central nerve reconstruction has been showing promising results through the development of an immunomodulatory and neuroprotective environment at the injury site [10-13] and has also been applied in various other experimental models of reconstructive surgery [7, 14]. The use of HFB has already been experimentally shown to provide a microenvironment of stability and protection at the reconstruction site, leading to positive scenarios of nerve regeneration. Therefore, this scoping review shed light on the association of HFB with sutures for nerve reconstruction following injury, highlighting its immunomodulatory and neuroprotective properties. Notably, integrating HFB with neurorrhaphy presents a promising avenue for surpassing the constraints of conventional sealants, while concurrently curtailing the extent of initial degeneration subsequent to injury.

## Methods

This scoping review follows the guidelines outlined in the Preferred Reporting Items for Systematic Reviews and Meta-Analyses (PRISMA) [15]. To achieve this, we conducted searches in PubMed/MEDLINE, Scopus (Elsevier), Web of Science, and SciELO databases, encompassing articles published until August 2023. The search used the keywords: “heterologous fibrin sealant,” “heterologous fibrin biopolymer,” “fibrin biopolymer,” “neurorrhaphy,” and “nerve injury” restricted to English-language publications.

To ensure precision, we carefully analyzed titles and abstracts to account for potential keyword overlaps. Articles were subsequently included or excluded based on predetermined eligibility criteria. Two independent authors conducted the selection process by using standardized procedures. When the title and abstract did not provide sufficient clarity, full-article analyses were undertaken. Review articles were scrutinized to identify potential experimental studies, and articles offering no novel insights were excluded following a comprehensive assessment.

**Figure 1.**
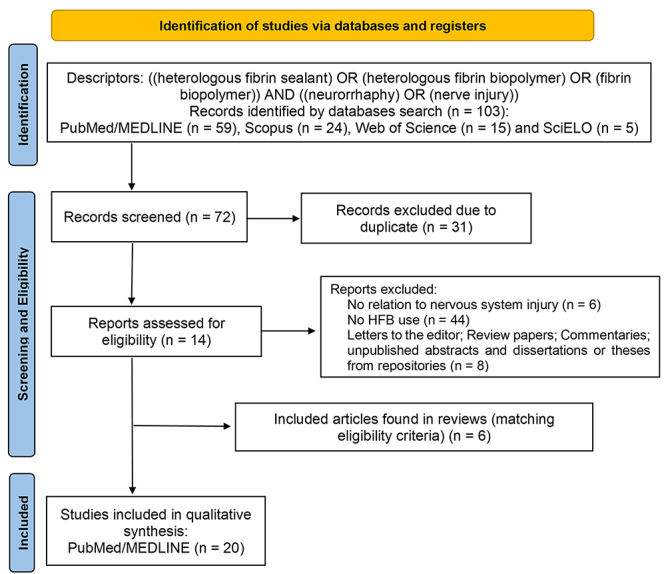
Flow diagram showing the study selection.

Among the initially identified 103 articles, 31 were eliminated due to duplication, and an additional 58 were excluded in line with eligibility criteria (not animal model study; no nerve injury model or nerve degeneration/regeneration involvement; no HFB use; not a research article). Notably, six research articles aligning with the stipulated eligibility criteria were identified through scoping and systematic reviews. A total of 20 articles regarding the use of HFB for nerve injury repair were then chosen for detailed text evaluation, which meets the predefined inclusion criteria, forming the basis for this review's scope (Figure 1). Furthermore, other articles were freely consulted to delve into further aspects of the biology of the neuromuscular junction, the pathophysiology of nerve injury, and the exploration of fibrin sealants for nerve injury repair. Table 1 summarizes experimental articles, which used HFB for neuronal regeneration included in this review.

**Table 1.** Summary of experimental articles involving neuronal regeneration (CNS and PNS) included in this scoping review.

Author	Objective	Methods	Results	Conclusion
Barbizan et al. (2014) [16]	To investigate two delivery strategies of mononuclear cells (MC), comparing the local injection to the spinal cord with the possibility of mixing MC with HFB on the interface of the CNS/PNS.	Forty female adult Lewis rats divided into: G1: avulsion only; G2: reimplantation with HFB; G3: root repair with HFB and MC; G4: root repair with HFB and injected MCs.	HFB enhanced cell therapy effects resulting in greater survival of spinal motoneurons up to four weeks post-surgery. MC added to the HFB increased neurotrophic factor gene transcript levels in the spinal cord ventral horn. The motor recovery was similar to cell-treated groups.	The use of HFB as a scaffold for the MC delivery approach gave the best and most long-lasting results. MC therapy was neuroprotective by increasing levels of brain-derived neurotrophic factor (BDNF) and glial-derived neurotrophic factor (GDNF).
Buchaim et al. (2015) [17]	To assess whether HFB permits the collateral repair of axons originating from a vagus nerve to the interior of a sural nerve graft and whether low-level laser therapy (LLLT) assists in the regeneration process.	Thirty-two adult male Wistar rats divided into: G1: intact sural nerve; G2: the ends of the sural nerve graft were coapted to the vagus nerve using HFB; G3: same procedures as G2 + LLLT.	The vagus nerve demonstrated collateral regeneration of axons to the interior of the autologous graft in all groups. G3 was similar to G1 concerning the area and thickness of the myelin sheath.	HFB + LLLT makes axonal regeneration feasible and is an efficient method to recover injured peripheral nerves, as well as improve myelination.
Cartarozzi et al. (2015) [18]	To investigate the effectiveness of mesenchymal stem cells (MSCs) associated with HFB for the peripheral regenerative process after nerve tubulization.	One hundred adult Lewis rats divided into G1: empty tube; G2: tube filled with HFB; G3: tube filled with HFB and grafted with MSCs.	G3 had a greater value for myelinated axon counting, more compact fibers, and a tendency to increase the thickness of the myelin sheath; with better motor function.	MSCs + HFB treatment was effective in improving nerve regeneration, as it positively modulated Schwann Cells reactivity.
Buchaim et al. (2016) [19]	To assess the impact of LLLT on the repair of the buccal branch of the facial nerve using two surgical methods: end-to-end epineural suturing and coaptation with HFB.	Forty adults male Wistar rats divided into: G1: suture; G2: HFB; G3: suture + HFB; G4: suture + LLLT; G5: suture + HFB + LLLT.	Axonal sprouting and morphology were similar among the experimental groups. G5 showed the closest results to the G1, in all measured variables, except in the axon area.	HFB allowed the coaptation of the stumps without trauma to the nerve fibers.
Rosso et al. (2017) [20]	To assess the impact of LLLT on facial nerve injuries repaired with the end-to-side method or coaptation with HFB.	Thirty-two adults male Wistar rats divided into G1: control; G2: suture; G3: HFB; G4: suture + LLLT; G5: suture + HFB + LLLT.	G4 and G5 groups showed higher mean values for histomorphometry and functional recovery variables.	The functional recovery of whisker movement occurred more rapidly in G4 and G5, with results closer to G1. LLLT expedited morphological and functional nerve repair in both techniques.
Spejo et al. (2018) [21]	To evaluate whether the combination of MSCs and HFB enhances the regeneration of the spinal cord following intramedullary axotomy (IA)	Eighty-eight adult female Lewis rats underwent a unilateral ventral funiculus incision at the L4, L5, and L6 spinal levels. The animals were divided into: G1: IA, G2; IA + vehicle; G3: IA + HFB; G4: IA + MSC; G5: IA + HFB + MSC.	MSC therapy: increased neuronal survival and functional recovery, reduced astrogliosis, and preserved spinal circuits. HFB promoted: early macrophage recruitment and expression of proinflammatory cytokines.	MSC therapy provides neuroprotection and, when combined with HFB, shifts the immune response towards the proinflammatory profile.
Mozafari et al. (2018) [22]	To determine whether human embryonic stem cells (hESC), either independently or in conjunction with HFBs, could aid in regeneration in a mouse model of sciatic nerve damage.	Forty-eight C57BL/6 J mice were divided into: G1: suture; G2: suture + HFB; G3: suture + HFB + doxycycline; G4: suture + HFB + wild type hESC; G5: suture + HFB + hESC off; G6: suture + HFB + hESC + doxycycline.	Sensory function was enhanced in G5, resulting in heightened reflexes, upon paw stimulation ipsilateral to the lesion, as evidenced by von-Frey evaluation, which was corroborated by immunohistochemistry.	Transgenic hESC could be used to support regeneration. HFB has the potential to aid nerve repair and is thought to promote superior functional recovery and improved motor neuron reinnervation when combined with this therapy.
Leite et al. (2019) [23]	To assess the effectiveness of HFB in association with suture, with 1 or 3 stitches after ischiatic nerve injury.	Thirty-six Wistar rats were divided into: G1: control; G2: denervated (6 mm gap); G3: 3 stitches suture; G4: 1 stitch suture + HFB.	G3 and G4 muscle analysis indicated an increase in muscle weight, frequency of fast fibers, and a decrease in collagen infiltration, while G4 had better nerve morphometric values compared to G3.	The findings imply a protective effect at the site of the lesion due to the use of HFB. The reduction in sutures lessens the trauma inflicted by the needle and expedites the surgical procedure.
Kempe et al. (2020) [24]	To determine whether the combination of pharmacological treatment with dimethyl fumarate (DF) and root reimplantation using HFB could promote neuroprotection, preservation, and recovery of motor function.	Seventy-nine adult female Lewis rats underwent ventral root avulsion of L4-L6 roots, followed by reimplantation and daily DF treatment for four weeks. HFB was utilized as an adjunct to ventral root reimplantation.	The association between HFB and DF preserved motoneurons and synapses, reduced astrogliosis and microglial reactions, and downregulated the expression of pro-inflammatory gene transcripts.	The association with HFB and DF demonstrated neuroprotective and immunomodulatory properties and 50% of motor function recovery following spinal cord root injury.
Pinto et al. (2021) [25]	To determine whether the combination of HFB with a single stitch has the potential to restore the function of the neuromuscular apparatus following sciatic nerve injury.	Forty Wistar rats divided into G1: Control; G2: Denervated (6 mm gap); G3: 3 stitches suture; G4: 1 stitch suture + HFB.	G1 exhibited normal morphology. In G2 flattening of the NMJ (fragmentation of nAChRs and tangled nerve terminals). G3 and G4: most parameter values were similar to G1 and G2. G3 and G4: planar area.	G3 and G4 showed less fragmentation of nAChRs, and protein expression values suggested a return of nAChRs to a mat The use of HFB in conjunction with a single suture stitch reduced surgical time, minimized suture injuries, did not affect nerve regeneration, and showed potential for restoring the NMJ apparatus.
Rodríguez-Sánchez et al. (2021) [26]	To determine if Polycaprolactone Nerve Guidance Conduits (PCL-NGCs) produced by 3D printing with canine Adipose-derived Mesenchymal Stem Cells (AdMSCs) embedded in HFB could enhance nerve regeneration after sciatic nerve injury.	Twenty-nine adult Wistar rats submitted to sciatic nerve injury were divided into G1: Sham; G2: autograft; G3: PCL (empty NGC); G4: PCL + HFB+ MSCs (NGC multi-functionalized with 10^6^ canine AdMSCs embedded in HFB).	G4 showed improved functional motor and electrophysiological recovery compared to the G3 after 12 weeks. G4 also increased the expression of neurotrophins and tended to higher reactivity of Schwann cells and axonal branching.	PCL + HFB+ MSCs promoted neuroregenerative effects and amplified the trophic microenvironment, resulting in a pro-regenerative state. These findings were associated with a shift in the regeneration process towards the formation of myelinated fibers.
Leite et al. (2023) [10]	To assess the impact of combining tubulization and HFB following peripheral nerve injury on the NMJ.	Fifty-two adult male Wistar rats divided into G1: control; G2: denervated; G3: tubulization; and G4: tubulization + HFB.	In G4 the NMJs presented morphological and morphometric similarities to G1 with an increase in S100 and AChRε protein expression and a decrease in MyoD.	The HFB association with tubulization resulted in nAChRs stabilization highlighting the beneficial effect of HFB when used in conjunction with the tubulization technique.
Tibúrcio et al. (2023) [13]	To assess neuroregeneration and the immune response in the context of neuromuscular recovery, using HFB in conjunction with suturing for rat sciatic nerve repair.	Forty male Wistar rats divided into groups of 7 or 30 days after nerve repair: G1: control; G2: denervated; G3: suture; G4: suture + HFB.	G4 had the highest M2 macrophage area in both periods. After seven days, the G4 showed an increase in nerve area, number, and area of blood vessels. In this period, G4 was the only one similar to G1 in terms of the number of axons. After 30 days, the G4 was closer to the G1 concerning blood vessels and central myonuclear numbers, NMJ angle, and connective tissue volume.	HFB enhances the immune response, increases axonal regeneration, induces angiogenesis, prevents severe muscle degeneration, and aids NMJ recovery.
Kempe et al. (2023) [27]	To examine the effects of combining surgical repair of lesioned roots with HFB and pharmacological treatment with dimethyl fumarate (DF).	Forty adult females divided into G1: reimplantation + vehicle; G2: VRA without reimplantation + DF; G3: reimplantation + vehicle + HFB; G4: reimplantation + HFB + DF.	HFB + DF association demonstrated: nerve sprouting, restoration of the ‘G’ ratio, and that most of the alpha motoneuron synapses were preserved in clusters. These parameters were associated with up to 50% of gait recovery, as the walking track test observed.	Combining root restoration with HFB and DF administration proved to be neuroprotective and can enhance motoneuron survival and regeneration after proximal lesions.
Bueno et al. (2023) [24]	To investigate the application of HFB in the repair of the buccal branch of the facial nerve (BBFN) in conjunction with photobiomodulation (PBM), using a low-level laser (LLLT).	Twenty-one rats divided into: G1: control; G2: LLLT; G3: denervated; G4: denervated + LLLT; G5: suture + HFB; G6: suture + HFB + LLLT.	G5 and G6 presented normal parameters related to functional analysis. G6 showed an increase in nerve fiber diameter and axon diameter compared to G6. In terms of muscle fiber area, G6 was similar to G1.	HFB+LLLT had positive effects on the morphological and functional stimulation of the BBFN, presenting a favorable alternative for the regeneration of severe injuries.

## Neuromuscular interaction

Nerves are formed by bundles of axons or nerve fibers, which are long and slender projections of a neuron that typically conduct electrical impulses, connecting different parts of the body to the central nervous system (CNS). Sensory signals are transmitted to the CNS through sensory nerve fibers (afferents), and the resulting response is carried by motor nerve fibers (efferents) to the target organs. Thus, a mutual communication pathway is established between the CNS and the peripheral areas of the body [1].

Among the motor nerve fibers, there is the lower motor neuron, a specialized cell with a highly developed dendritic arborization. Its cell body is located in the ventral column of the gray matter of the spinal cord, and its axons extend throughout the body via peripheral nerves until they reach the target organs [28, 29], the skeletal striated muscle, where it forms a morphologically, molecularly, and functionally specialized region called NMJ. At this site, there is transmission and transduction of an electrical signal (nerve impulse) and a chemical signal (neurotransmitters) from the motor neuron to the muscle fiber, promoting muscle contraction. This junction presents a major subclass of synapses in the mammalian nervous system, critical for the transfer of information between the lower motor neuron and the skeletal muscle. It also represents a conveniently accessible "model" synapse within the peripheral nervous system [29]. 

## Neuromuscular junction organization

**Figure 2.**
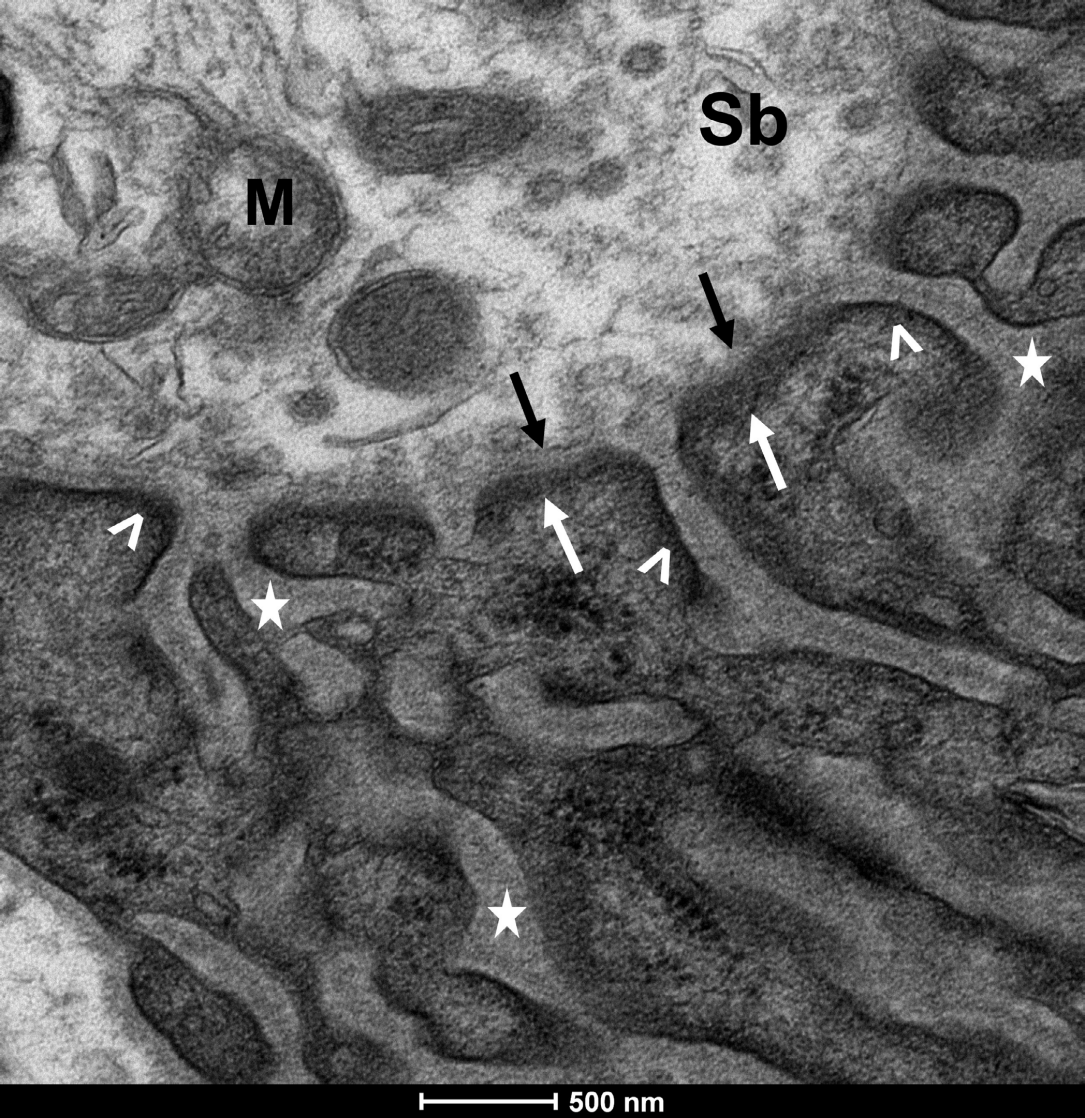
Components of the neuromuscular junction. Transmission electron microscopy image of a Wistar rat soleus muscle NMJ. Sb: Synaptic button; M: mitochondria; →: ACh vesicle; *: junctional folds; >: nAChRs cluster; Black arrows: presynaptic membrane; white arrows: postsynaptic membrane.

The NMJs are small structures (30-60 um) when compared to the length of innervated muscle fibers, and each skeletal muscle fiber is recruited by a single NMJ [30]. The classic morphology of the NMJ, first described in rodents, is characterized as a "pretzel" shaped structure, and changes in this structure are closely associated with motor pathologies [28, 30]. The most common classification of NMJs divides them into three closely associated cellular compartments (Figure 2).

### I - Presynaptic membrane (nerve terminal)

The terminal branches of lower motor neuron axons form synaptic boutons, hosting synaptic vesicles carrying the neurotransmitter acetylcholine (ACh). This nerve terminal holds various cellular constituents, including mitochondria, endoplasmic reticulum, lysosomes, organic substances, and metal ions [31, 32]. Terminal or perisynaptic Schwann cells (tSCs) are found in this region [33-35], they are non-myelinating glial cells whose extensions cover the nerve terminals, protecting them from chemical and mechanical injuries [36]. Additionally, tSCs are the main cells responsible for the structural plasticity of NMJs by perceiving possible alterations in synaptic transmission [37, 38]. One to three tSCs surround the nerve terminal, and actively participate in the development, maintenance, and repair of synaptic function [30], in addition to producing a basal lamina that maintains the structure of the NMJ [39].

### II - Synaptic cleft

The synaptic cleft separates the presynaptic nerve terminal from the postsynaptic membrane by a narrow gap of 50 to 80 nm in width. It is filled with the synaptic basal lamina, composed of molecules secreted by both the nerve terminal and the associated muscle fiber [40]. This region houses crucial proteins for NMJ stability, function, and upkeep, including those responsible for the clustering of nicotinic acetylcholine receptors (nAChRs) on the sarcolemma, such as agrin, laminins-4, 9, and 11, matrix metalloproteinase-3 (MMP3), and collagen IV. Acetylcholinesterase (AChE), essential for degrading ACh post-muscle contraction, is also concentrated here [41, 42], culminating in the termination of neuromuscular transmission [43].

### III - Postsynaptic membrane

The postsynaptic membrane compromises the plasma membrane of the muscle fiber with junctional folds, where the nAChRs and the sarcoplasm of the muscle fiber (cytoplasm) are located [34, 35]. These folds exhibit two distinct regions: the crest, enriched with nAChRs and additional proteins like rapsyn, utrophin, and α-dystrobrevin-1 to ensure stability; and the trough, hosting α-dystrobrevin-2, dystrophin, NCAMs for muscle integrity and axonal guidance. Alongside these, voltage-dependent sodium channels responsible for generating action potentials are also present [43-45]. The sarcolemma's invaginations amplify the postsynaptic membrane's surface area and facilitate the juxtaposition of nAChRs with the presynaptic zones, while sodium channels extend within the sarcoplasm. Basal lamina encompassing extracellular matrix components tightly enwraps the muscle fibers, connecting with the basal lamina of the synaptic cleft produced by tSCs at the NMJ's periphery [43].

At the NMJ, nAChRs are the primary receptors in the muscle to establish neuromuscular motor communication [46], thus maintaining and facilitating synaptic transmission. Structurally, they comprise five subunits arranged in a rosette pattern, which collectively form ion channels [47]. These receptors are distributed in two forms: immature extrajunctional, present in embryonic muscle fibers, composed of subunits two alpha (α), beta (β), delta (δ), and gamma (γ); and mature junctional, in which α (2), β, and δ subunits are maintained, and the γ subunit is replaced by epsilon (ε) [31]. 

Activation of nAChRs is initiated by the simultaneous binding of two ACh molecules or other agonists at the juncture of the αδ and αε subunits, thereby inducing a conformational alteration that opens the ion pore and triggers its active state [48, 49]. This active state's duration is modulated by the interaction between the receptor and ACh molecules, promptly broken down by AChE, returning nAChRs to their quiescent configuration [49]. In instances of injury or pathology, nAChRs often revert to an embryonic pattern, featuring the gamma subunit instead of epsilon [50]. This exchange of γ- for ε-subunits contributes to several changes in stability, kinetic, and transmission properties [28]. There are also positive and negative signals involved in the maintenance of the NMJ, and fundamentally, nAChRs clusters [30]. The main positive signal is the binding that occurs in the Agrin/LRP4/Musk/Rapsyn pathway [25].

## Pathophysiology of nerve injury

Following nerve transection, a cascade of physiological and metabolic responses is triggered at the cellular level in the injury site and in the cell body of the affected neurons in an attempt to stabilize the damage resulting from the injury [28]. In neurotmesis, the nerve is divided into two stumps, distal and proximal to the site of injury, which are subject to relatively distinct processes [51]. Shortly after, the process of Wallerian degeneration (WD) begins in the distal stump, while the proximal stump changes its gene expression profile, adopting a repair profile, inducing degenerative changes followed by a regenerative process [30]. Shortly after axotomy, axons from the proximal stump undergo a process of swelling, characterized by a reorganization of the cytoskeletal structure to seal themselves through a fibrotic cascade to contain leakage of the axoplasm [3].

In the distal stump, where axons lose contact with neuronal bodies, Wallerian degeneration takes place, manifesting as myelin breakdown and axonal disintegration. This process initiates with a rapid influx of intracellular calcium, triggering protease activation and granulation within the axoplasm through the proteolysis of microtubules and neurofilaments. This succession of events further undermines the organizational integrity of the cellular structure [51, 52]. Within this milieu, damage-associated molecular patterns (DAMPs) and pro-inflammatory agents, including tumor necrosis factor (TNF-)α, interleukin (IL-) 1β, and CCL2, activate the initial immune response, mostly attributed to macrophages [53]. One of the primary functions of these macrophages is the clearance of tissue debris, a role reinforced by Schwann cells through myelin autophagy [54]. It is important to note that Schwann cells, fibroblasts, and resident macrophages also contribute to the inflammatory milieu by releasing proinflammatory mediators [55]. 

Furthermore, additional components of the immune system have been associated with effective Wallerian degeneration, often through their modulation of the macrophage-mediated response. Neutrophils are notably the first subset of cells to infiltrate the injured nerve, driving initial tissue clearance and recruiting monocytes from the peripheral circulation, which subsequently differentiate into macrophages [55]. Complement proteins and antibodies present in the serum enhance macrophage attraction and response by opsonizing cellular debris, thereby facilitating phagocytosis. The last cells to participate in the immune response within the damaged nerve are T lymphocytes [55, 56]. While recognized for their role in the amplification and regulation of the inflammatory response, their specific function in nerve regeneration remains underexplored. It’s worth mentioning that nerve regeneration has traditionally been correlated with distinct macrophage subsets: pro-inflammatory type 1 macrophages (M1) driving the degenerative phase, and anti-inflammatory type 2 macrophages (M2) guiding the regenerative phase [57]. These subtypes undergo polarization in response to cues from the degenerative or regenerative environment, yielding unique phenotypes, metabolic traits, and functions [57]. However, classifying macrophages solely into M1 and M2 categories oversimplifies the intricate cell landscape within the nerve, where an array of macrophages expressing various pro- and anti-inflammatory markers coexist.

Within tissue clearance, regeneration slowly begins at the proximal end of the injury site and proceeds toward the distal segment. In this context, Schwann cells close to the damaged region dedifferentiate, proliferate, and migrate to the lesion site. There, they align up within endoneurial tubes, forming longitudinal columns known as bands of Büngner, inside of which they release growth and trophic factors to stimulate axonal elongation up to the target [5]. Importantly, the correct orientation of Büngner’s bands depends on a polarized vasculature, induced by macrophage-derived VEGF-A upon hypoxia stimulus. Those newly formed blood vessels are essential for regeneration as they serve as tracks, guiding Schwann cell migration across the wound and subsequent axonal elongation [58].

In the proximal stump, neurotmesis evokes a retrograde response in neuronal cell bodies [59]. Surviving neurons undergo a series of physiological and morphological changes called chromatolysis, which is characterized by swelling and rounding of the cell body, dissolution of Nissl granules, and eccentric displacement of the nucleus and endoplasmic reticulum [1, 60]. These structural modifications are paralleled by a shift in physiological cellular metabolism towards the synthesis of proteins involved in axon regeneration [59], such as those related to the cytoskeleton and growth factors [51]. With the elimination of myelin debris, regeneration slowly begins at the proximal end of the injury site and proceeds toward the distal segment. The severed axons produce a large number of sprouts, closely associated with Schwann cells, forming several growth cones in the distal direction. 

On the other hand, the distance of the injury from the cell body implies a worsening of the regenerative condition, as observed in sensory neurons, where the closer proximity to the dorsal root ganglia is directly related to the number of dead nerve cells [61] and a greater effective distance for regeneration. Strikingly, although axons do regenerate, studies have demonstrated that the morphological recovery of the nerve and muscle is not definitive for functional recovery, as there may still be impaired synaptic functionality [62, 63]. 

**Figure 3.**
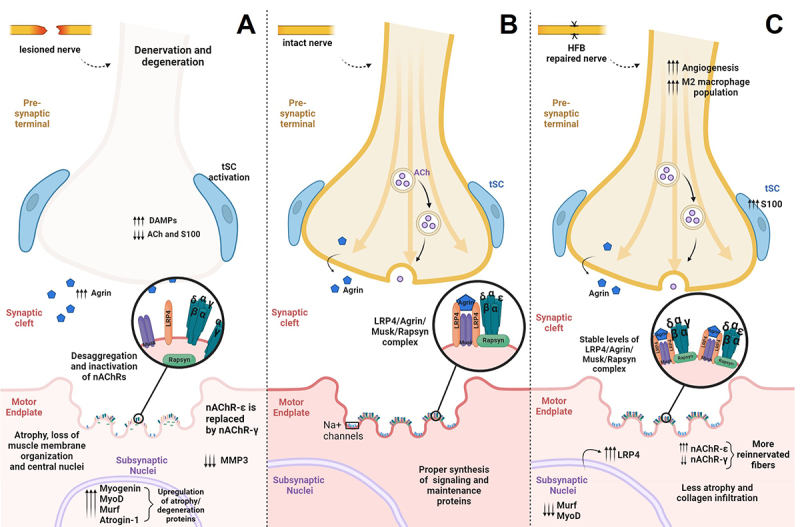
Cellular and molecular events associated with muscle denervation and repair at the neuromuscular junction level. **(A)** Denervated, **(B)** healthy, and **(C)** HFB repaired neuromuscular junction. Following nerve injury, all components of the NMJ are hindered and the Agrin/LRP4/Musk/Rapsyn signaling pathway plays a significant role in the nAChRs cluster formation and NMJ maintenance.

Following the injury, nerve endings that formed NMJs are lost, and even with successful axonal sprouting, the failure of reinnervation limits muscle function recovery [64] (Figure 3A). Irregular nerve branching, poly-innervation [65], and collateral reinnervation of muscle fibers commonly occur. Denervated NMJs suffer severe degeneration and subsequent disintegration [66] and disassembly of their nAChRs and anchoring proteins, which are fundamental for their function maintenance [67]. When axons are damaged, they release DAMPs that activate tSCs. In turn, these tSCs release chemokines, which serve as guidance substrates, facilitating the formation of tSCs bridges and thus promoting NMJ reinnervation [28, 39]. The nAChR-ε subunit is replaced by nAChR-γ, altering the transmission of the electrical signal leading to muscle failure and atrophy [68], as ACh is also no longer available at the synapse.

Denervation also leads to significant changes in the Agrin/LRP4/Musk/Ra pathway, prejudicing the maintenance of nAChR clusters [69] and leading to alterations of the area and conformation of the NMJ [10]. Shortly after injury proteins of this pathway have increased synthesis but its complex disorganization leads to failure in proper function, inactivation of MMP3, and accumulation of Agrin in the synaptic cleft [28]. The NMJ microenvironment is also deprived of other retrograde and anterograde signals that maintain its stability [28], in which activated tSC extend their processes synthesizing nAChR in non-synaptic muscle regions. 

In the regenerative process, the Agrin protein has a fundamental role in initiating the receptor clustering pathway, remodeling, and returning the NMJs to their conventional shape [30]. The LRP4 protein, dependent on Agrin for activation [69], also has a fundamental role in the regenerative process, contributing to the activation of tSCs after injury, which is responsible for guiding the reinnervation of NMJs in denervated fibers [10]. Notably, active tSCs also maintain NMJ organization by secreting substrates that regulate postsynaptic proteins, such as MMP3, which is involved in basal lamina and endplate integrity [66, 70]. In the maintenance of postsynaptic structures, other muscle-related proteins play a crucial role, as it has been demonstrated that the prevention of denervation is associated with the increased expression of MuSK, Dok7, and those involved in the ubiquitin-proteasome pathway, such as MURF-1 and atrogin-1 [10, 28]. The shift of gamma to the epsilon subunit in the nAChR serves as an indicator of regeneration, as it occurs only after reinnervation and differentiation of the myotube.

Altogether, major cellular and molecular events take place and deprive not only the associated nerve, Schwann cells, and muscle fibers but also the endplate, eliciting a series of retrograde and anterograde signals that, even with morphological regeneration of axons, can result in no definitive association with functional recovery. Understanding the therapeutic niche in the NMJ, as an end-gate player, is essential to provide positive outcomes after reinnervation, which includes not only reinnervation through the arrival of regenerating axons to denervated postsynaptic muscle domains but also its capability of synaptic activation (Figure 3B). 

## Approaches to peripheral nerve repair

In recent years, several approaches have been tested and used to restore functionality to the injured nerve [71, 72]. Most experimental models use rodents performing surgical lesions of the facial, ulnar, or sciatic nerves [73]. Treatments that result in complete morphologic and functional recovery are still a challenge for medical-surgical practice. The main difficulties are related to nerve reconnection and regeneration itself, in which regenerating axons fail to select their original endoneurial tubes [51], local revascularization, prolonged period of injury without medical intervention, progressive loss of regenerative character in Schwann cells, and atrophy of the innervated organs [51, 74-76].

Among the techniques for correcting nerve transection, end-to-end neurorrhaphy is the gold standard [46]. This type of neurorrhaphy is only possible when the nerve stumps are preserved, visible, and intact, without tissue loss, and there is no tension for re-approximation and reconnection of the stumps [11]. When such favorable characteristics for reconnection are not present, other techniques are used, including end-to-side neurorrhaphy [77], grafts and conduits [10, 22], and the use of tissue repair adjuvants such as fibrin sealants [78] and photobiomodulation [79].

Over the years, suture materials and techniques have presented problems and limitations, which motivated research on adhesive materials that can bond tissues. The first research on hemostatic and adhesive agents began around 1940, during World War II when fibrin glue was proposed. The fibrin glues or sealants, which can connect tissues more quickly, are important as they promote tissue adhesion and hemostasis [7]. At that time, a mixture of human fibrinogen and thrombin was mainly applied to areas of skin affected by war wounds. The structural characteristics of fibrin(ogen) can be encapsulated by the processes of fibrin polymerization and cross-linking. These processes facilitate a multitude of biological functions, which include, but are not limited to, thrombin binding, fibrinolysis, the control of coagulation protein activity, the binding of growth factors, and interactions with various cells [80].

In the 1970s, the concept of fibrin glue was re-evaluated, leading to the introduction of the first commercial sealant, *Tisseel* (Baxter International, Inc., Deerfield, IL), containing human fibrinogen and bovine thrombin. These sealants have been successfully marketed for years [72, 81, 82]. However, its commercialization was suspended and its use was prohibited in the USA by the Food and Drug Administration (FDA) agency in 1978, due to the risk of transmission of infectious diseases conveyed by human blood-derived products [7, 82]. In the early 1980s, the discovery of the human immunodeficiency virus added credibility to the FDA's position, which feared widespread viral transmission [8]. However, in May 1998, the FDA revoked the suspension and approved the clinical application of fibrin sealants in the USA, when they were once again marketed [8].

Fibrin sealants are adhesive substances that mimic the final stages of blood coagulation. They form a stable, physiological fibrin clot which aids in wound repair. In addition to this, they serve two other primary functions. Firstly, they provide physical support for the extracellular matrix. Secondly, they facilitate the delivery of treatment compounds. This is made possible by their capacity to maintain the biochemical factors and original properties of implants [80, 83-85]. Therefore, they reduce the risk of bleeding, allow for the use of fewer sutures, and protect the injured environment against infections by microorganisms [85, 86]. They also allow for a decrease in inflammatory reactions, a lower incidence of trauma due to sutures [1, 78], and faster surgery and recovery, due to easy and fast applications in emergency conditions, allowing for a non-experienced surgeon to perform the repair. Whitlock et al. [87] investigated experienced and novice surgeons regarding the speed and quality of nerve repair in rodents, and showed a shorter surgery time in both scenarios with sealant repair, instead of sutures, and still, equivalent quality in sealant repairs between surgeons, demonstrating a faster learning curve.

In general lines, fibrin sealants are produced in two ways: from autologous or homologous blood derivatives. Autologous sealants use the patient's blood. Although they are biocompatible and do not present a risk of transmission of infectious diseases, they are not viable in surgeries and emergency applications. As an alternative, homologous fibrin sealants, produced by a pool of human blood, have been used. However, in these cases, there are risks of transmission of infectious diseases such as hepatitis, HIV, and human parvovirus [14]. In addition to having a high cost of raw materials, with a need for blood extraction of bovine or human thrombin and human fibrinogen. Due to their composition with human blood, another common problem is rapid fibrinolysis, which starts less than 24 hours after application and prematurely detaches the nerve stumps [14].

Even with its not-so-recent introduction in human clinics, there is still no consensus on the use and efficacy of fibrin sealants for nerve repair, with a predominance in the literature of studies with rodent models, and few evaluations in humans [9, 78]. The use of sealants is still not approved by regulatory agencies such as the FDA for nerve repair, but the interest in this therapy, not only for nerve reconstruction, is growing. In a study with American surgeons, Owosu et al. [88] showed that a portion of them already use or consider using fibrin sealants in repairs, although there is still a preference for suturing as the main repair technique. The lack of data on the results and knowledge of the treatments were identified as the main barriers to the use of adjuvant therapies. However, most surgeons are open to the possibility.

Regarding efficiency in nerve repair, several studies show beneficial effects associated with the application of sealants, alone or with sutures, at the site of the injury. A systematic review conducted by Sameem and collaborators [78] supports, with the majority of studies in rodents, an equal, if not superior, performance in repair with fibrin sealants compared to micro-sutures only, based on histopathological, biomechanical, and electrophysiological characteristics. However, in treatments with sealant only, cases with difficulties such as complete failure and gaps in neural reconnection were also observed. A recent systematic analysis [9] indicates that nerve regeneration may be similar in fibrin glue repairs and/or suture repairs, but the use of fibrin glue significantly reduced operation times, which may pose both clinical and economic benefits. However, fibrin glue alone was reported to result in lower strength and more dehiscence [9, 89].

Until today, commercially available fibrin sealants are produced from both bovine and human thrombin and fibrinogen (homologous), which present disadvantages and risks, so that in addition to being transmitters of infectious diseases, they can generate fibrosis, toxicity, and necrosis [1]. They can also lead to the development of antibodies against bovine thrombin, and anaphylactic reaction to bovine proteins, among other adverse reactions [7, 82].

## HFB in nerve repair

Considering the disadvantages present in commercially available fibrin sealants, a group of researchers from the Center for the Study of Venoms and Venomous Animals (CEVAP) at São Paulo State University, Botucatu, São Paulo, Brazil, began to standardize a new fibrin sealant in the 1990s. The HFB is a unique heterologous fibrin worldwide, as it does not use human blood, and does not transmit infectious diseases. In addition, it is a natural, biodegradable, bioabsorbable, and non-toxic biopharmaceutical with excellent adhesive capacity, and can be used in several surgical procedures.

Compared to other clinically used commercial fibrin sealants, it has a low production cost, high availability of raw materials for its manufacture, and the possibility of adapting its formulation according to the type of procedure [82]. Its formulation allows its use in clinical medicine and various surgical procedures, such as skin grafts and surgical reconstructions, aiding in hemostasis, colostomy, and suture reinforcement [11, 14]. Furthermore, its healing power was tested in 40 patients of a phase I/II clinical trial for the treatment of chronic venous ulcers. Clinical results showed it as a safe, non-immunogenic product with promising efficacy. This is because there was an improvement in the quality of the ulcer bed, a significant reduction in the ulcer area, and healing in several patients [90, 91].

**Figure 4.**
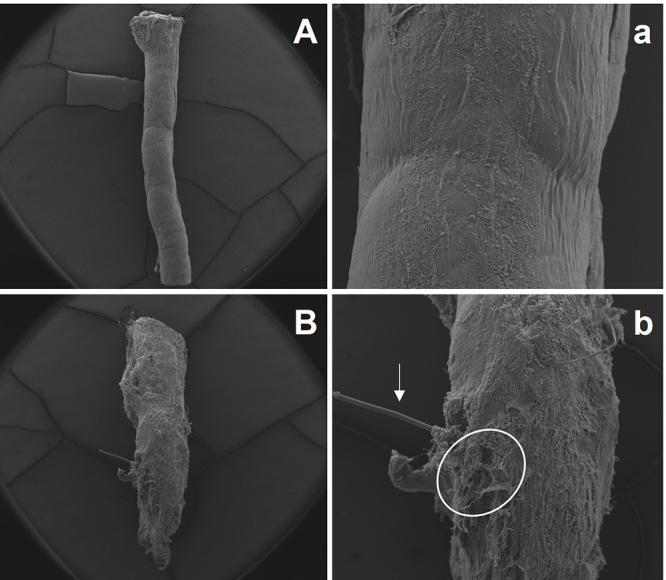
Nerve integrity of healthy and HFB-repaired nerves. Scanning electron microscopy of **(A)** healthy and **(B)** HFB-repaired nerves. Surface detail of epineurium can be seen in **a** and **b**. White arrow: nylon suture thread; white circle: HFB-associated connective tissue.

In the last years, HFB has been used in association with sutures (Figure 4) and other therapeutic factors to enhance or face difficulties in the current gold-standard treatment for nerve injury. Generally, in the face of a nerve transection, the application of HFB demonstrated positive results in reducing mechanical trauma to the nerve (points of suture) [23], obtained better or similar morphological and/or molecular performance when adjunct to traditional suture [7, 13, 23, 25], demonstrated easy usability with good adhesive capacity [17], and good biocompatibility with other materials [26, 92] (Figure 3C). Its healing power has been gaining attention and demonstrated valuable potential as an adjunct to enhance regeneration and motor function (Table 2). 

**Table 2.** Current strategies and potential therapeutic strategies aligned to HFB use for traumatic peripheral nerve injury.

Strategies (neurorrhaphy)	Intervention	Comments
Alone [13, 17, 19, 20, 23, 25, 93]	HFB as an adjunct to 1 to 3 stitches	HFB demonstrated angiogenic, neuroprotective, and immunomodulatory properties, which can enhance tissue regeneration, motor function, and wound healing, but satisfactory outcomes have not yet been achieved. The use of HFB also reduces trauma and the number of stitches.
Nerve conduit [10, 18, 26]	HFB embedded tubulization with suture	This association suggests a trophic action of the treatment, potentially accelerating regeneration through an increase in regeneration-associated factors, such as those related to Schwann cell reactivity (S100) and Agrin/Rapsyn/LRP4/Musk signaling pathway for the NMJ maintenance. Polycaprolactone has demonstrated good biocompatibility with HFB, surpassing other fibrin sealants.
Cell-based Therapy [16, 18, 22, 26, 94-96]	HFB as a scaffold for Embryonic Stem Cells, Mesenchymal Stem Cells, and Mononuclear Cells	HFB improves the effects of cell therapy by promoting an environment that enhances each particular scenario, such as growth factors release, and increased cell survivability resulting in neuroprotection.
Photobiomodulation [17, 19, 20, 93]	HFB as an adjunct to Low-level laser therapy with and without suture	HFB in association with LLLT has demonstrated a strong capability to accelerate myelination and functional recovery of innervated muscles. HFB allows nerve manipulation without trauma and enhances the nerve regeneration process.

### Adjunct to suture alone

**Figure 5.**
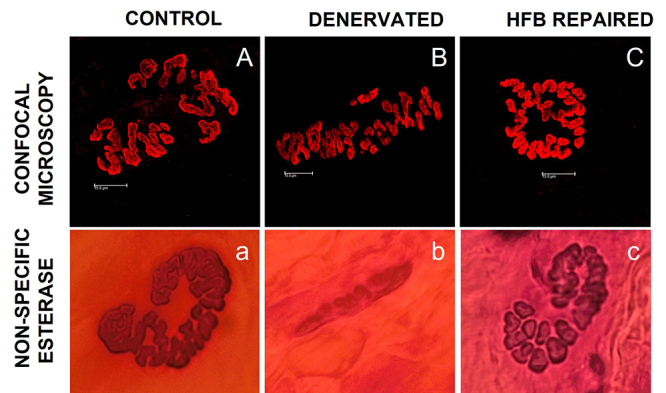
Neuromuscular junction morphology. **(A, a)** Healthy, **(B, b)** denervated, and **(C, c)** HFB repaired states, through nAChRs staining (confocal microscopy) with alpha-bungarotoxin conjugated with rhodamine and by non-specific esterase staining, which is based on the marking of positive esterase sites that contain AChE, leading to visualization of the entire NMJ morphology [97].

For short nerve injury gaps (< 1 cm), neurorrhaphy is commonly employed, repairing both proximal and distal ends, but the use of sutures can lead to inflammatory reactions such as granuloma and neuroma formation. In the last years, our group has been studying the effects of combining HFB with neurorrhaphy, also shading light to the NMJ recovery (Figure 5). HFB plays a crucial role in the healing process as the combination of fibrin with proteins enhances angiogenesis, wound contraction, collagen synthesis, and re-epithelialization. Further, it was able to reduce trauma in reconstruction to minimize the damage and inflammatory responses.

In a recent study, we demonstrated the early beneficial effects of the use of HFB in conjunction with two sutures for the repair of transected sciatic nerves in rats [13]. HFB application in comparison to suture alone improved overall nerve regeneration, with increased axonal growth and tissue vascularization (angiogenesis), proper restoration of neuromuscular junctions, reduced severe muscle degeneration (collagen infiltration), and enhanced relative area of M2 markers, 7 and 30 days after reconstruction. Remarkably, macrophages play a significant role in phagocytosis and cytokines releasement in the denervated NMJ immune response, and its balance between pro- and anti-inflammatory effectors at different phases of NMJ reinnervation appears to be crucial [64].

Sixty days after nerve reconstruction [23], with a 7-day interval between sciatic nerve injury and neurorrhaphy after 60 days of reconstruction, the association of HFB with one suture point showed restoration of nerve impulse and better axonal regeneration than suture alone. Ultra-structurally, the NMJs and associated muscle fibers of the HFB-treated groups showed proper regeneration. In this same study design [25], the use of HFB associated with a single suture point, compared to two suture points, reduced surgical time and showed potential to restore the microstructure of NMJs, as less degeneration was present and nAChRs/proteins associated with the mature pattern were observed to return. In addition, immature receptor values (γ) in the HFB group were lower than those in the suture-only group.

### Multimodal approaches (cell-based therapies and nerve conduits)

Another therapeutic option for nerve injury is the use of nerve conduits, which serve as a bridge between the proximal and distal stumps, providing a scaffold for axonal regeneration [1]. This approach prevents intrusion of nearby tissues, guides the regeneration, and makes the repair site less prone to infiltration by fibroblasts and adverse inflammation, which may decrease fibrosis and improve overall nerve mobility [3]. HFB has already been associated with nerve conduit strategies and has demonstrated good biocompatibility with the polycaprolactone (PCL) grafts, which has been a challenge for other commercially available fibrin sealants [26], improving general regeneration (Table 2).

Our group used a PCL graft in addition to one suture point associated with HFB [10], and after 90 days of nerve reconstruction, although not fully regenerated, as indicated by the nAChR cluster area, the compactness and endplate area of NMJs in the treated group were the only similar the Control, suggesting a better morphological approximation between the groups. Notably, it also demonstrated an increase in the expression of LRP-4, S100, and nAChR-ε proteins, and a decrease in MyoD, suggesting a positive influence on the neurodegenerative process, where the LRP-4 activates tSC, thus maintaining higher reinnervation of nAChRs and reducing muscle atrophy. The treatment favored a faster recovery in motor function assessment by improving print area when compared to suture alone. Indeed, the nerve guidance with HFB also enabled an approximation to the control group in terms of protein expression of Agrin, LRP-4, Musk, and Rapsyn.

Another promising augmentation method is cell-based therapy, which is a way of enhancement that can speed up the self-healing process and is currently under extensive research for stimulating regeneration after nerve injury [98]. The therapy employs stem cells due to their inherent ability to self-replicate and differentiate into specific cell types [1], which culminates in the release of neurotrophic growth factors and the myelination of axons. Schwann cells, the primary functional cells of the peripheral nervous system that promote myelination and regeneration, are the preferred initial seed cells, but other cell types have also been used and have achieved remarkable results [99]. For successful cell transplantation and adhesion at the injury site, an appropriate scaffold is necessary. HFB has demonstrated good biocompatibility, while further enhancing the power of regeneration-associated factors of the cell lineage and also improving the survival of the cells (Table 2).

The first study utilizing this association was conducted by Mozafari et al. [22], who observed axonal regeneration and sensory function improved using HFB with embryonic stem cells in a rodent model 60 days after sciatic nerve injury, where the combined effect was successful in supporting Schwann cells at the injury site and HFB facilitated the application and stabilization of the stem cells. In another study, Rodrigues-Sanchez et al. [26] used HFB as a base for PCL graft and canine adipose mesenchymal stem cells, observing that this multimodal approach supports the trophic microenvironment, resulting in a pro-regenerative state after critical sciatic nerve injury in rats. The treatment incorporated in HFB demonstrated enhanced motor function and electrophysiological recovery compared to the PCL group after 12 weeks. These results were linked to a change in the regeneration process favoring the development of myelinated fibers. HFB is demonstrated to be very permissive for use in conjunction with stem cells, allowing for an even more efficient regenerative process [92]. Further, Cartarozzi et al. [18] observed similar results engrafting HFB with mesenchymal stem cells in PCL conduits, where this association resulted in better reactivity of the glial cells leading to better regeneration and compacting of myelinated axons, as well as functional improvement in gait recovery. It was also observed that HFB enabled the survival of the cells seeded in the tubular prosthesis.

### Photobiomodulation

Also known as LLLT, photobiomodulation is an emerging therapy that is increasingly being used for rehabilitation and functional restoration following injuries. The therapeutic effects of LLLT are linked to tissue biostimulation. This therapy triggers photoenergetic and photochemical reactions, leading to an increase in DNA and RNA synthesis within the cell nucleus [100]. In turn, this promotes cell proliferation and protein synthesis, including alterations in the action potential of nerve cells. For the treatment of nerve injuries, it has been reported that this therapy stimulates myelination and axon regrowth by promoting Schwann cell proliferation [101]. In recent years, this technique has been studied in association with HFB, which has shown promising results, as HFB minimizes trauma and together exhibits strong regenerative power.

The initial study investigating this association was carried out by Buchaim et al. [17]. This study, which also involved a nerve graft, noted the collateral regeneration of axons from the vagus nerve into the autologous graft in all groups, but HFB+LLLT had enhanced myelination. In a subsequent study, Buchaim et al. [19] reported a comparable improvement in axonal recovery of the facial nerve post-suturing with the HFB-repaired group, which showed the closest results to the control in all nerve measurements. Additionally, the use of HFB facilitated the coaptation of the stumps without causing trauma to the nerve fibers. Further, Rosso et al. [20], also in a facial nerve model, observed better morphofunctional results in sutures or coaptation with HFB associated with LLLT, with an advantage in reducing trauma in the HFB group. In another recent study, this approach improved axonal growth in the stump distal to the lesion and minimized the atrophy on innervated muscles after experimental facial nerve transection [93]. HFB+LLLT had positive effects on the morphological and functional stimulation of the nerve, with the greatest regeneration for axon area and diameter.

Photobiomodulation and HFB were also assessed in other regenerative approaches, such as in bone, tendon, and skin repair [102-104]. All these investigations have revealed positive outcomes, underscoring the potential enhancement achievable through the synergistic use of HFB with other therapies. Unfortunately, no data was obtained regarding the NMJ regeneration process.

### HFB in spinal cord injuries

Due to the proximity between the CNS and PNS interface, learnings can be made for the treatment of nerve injuries based on the use of HFB in CNS lesions, which has also been a hot topic approach. Spinal cord injuries result in a significant loss of motor and sensory functions. Ventral root avulsion, an experimental model, involves the detachment of the ventral (motor) roots from the spinal cord’s surface. This leads to numerous morphological alterations, including the degeneration of motoneurons and rearrangements in the local spinal cord circuitry. Further, it has several commonly observed pathologies for nerve injury, such as WD.

In a rat ventral root avulsion model, Barbizan et al. [12] demonstrated that the root replantation with HFB enhanced motor recovery preserved the synaptic covering of the motoneurons, and improved neuronal survival. Barbizan et al. [16] further demonstrated that ventral root avulsion repair with HFB as a scaffold for mononuclear cells enhanced motoneuron survival and neurotrophic factor expression levels. In the same model, Spejo et al. [21] reported a potential immunomodulatory effect with increased expression of M2 macrophage marker genes and pro- and anti-inflammatory cytokines, along with greater neuronal survival when combined with HFB reconstruction. Kempe et al. [24] demonstrated a 50% increase in motor function, neuronal and synaptic survival, and immunomodulatory effects with the use of HFB, compared to its absence when combined with dimethyl fumarate after ventral root avulsion. Kempe et al. [27] further demonstrated the preservation of alpha motor neuron synapses and survivability of its associated sciatic nerve with spouting and restoration of proper myelination and axon diameters by the combined treatment through ultrastructural evidence. Paes et al. [105] used HFB as a scaffold for human dental pulp stem cells for reimplantation after ventral root avulsion and also observed higher neuronal survivability and downregulation of glial reactivity, while also enhancing motor function in catwalk analysis.

All those works, for both peripheral and CNS injuries, highlight the HFB potential as an excellent scaffold for adjunct stem cell therapy and drug delivery systems. Indeed, the treatment of motor neuron injuries with HFB demonstrated improvement in tissue regeneration and motor function, although, in a different environment, the axon regeneration process was also enhanced.

### Limitations and difficulties

Based on these presented studies and current strategies, which investigate the HFB association with nerve regeneration, further combinations with other therapies, such as regeneration-associated factors (growth factors), may enhance positive benefits. HFB readily allows for the encapsulation of bioactive complexes in its formulation, including growth factors, cytokines, drugs, and nucleic acids, which may further improve the therapy effect. Further, although remarkable results have been achieved with cell therapy, no studies have been conducted using Schwann cells, which are the most studied therapeutic model for nerve injury. 

In relation to HFB volume, there is no current consensus on when it is used in nerve repair. The values range from 100 µL (2 drops) to 500 µL (10 drops). Despite this variability, it is believed that the healing effect, or the support provided by the scaffold therapy may not be compromised.

Regarding NMJ regeneration, no data was found on its association with cell therapy and photobiomodulation. Studies of the NMJ are crucial to understanding the sequence that extends from the central stimulus to the nerve-muscle tissue targets; this is particularly important as most therapies, while offering certain morphologic benefits, also present limitations related to motor function. The neuromuscular plasticity observed following nerve injuries and the subsequent reinnervation of muscles is remarkable, however, this may not be sufficient to restore fine movements due to the considerable misdirection of regenerating nerve fibers and failures or insufficiencies in NMJ reinnervation following injuries. 

Another challenge lies in the development of pharmaceuticals that adhere to good manufacturing practices and produce consolidated, reproducible batches. To address this, CEVAP is launching the first Brazilian Contract Development and Manufacturing Organization (CDMO) [106]. This organization will provide services to both the public and private sectors by producing validated samples for clinical trials and offering academic training in translational science research. Furthermore, CEVAP is currently seeking support to conduct a Phase III multicenter clinical trial to validate the HFB findings, register with the Brazilian Health Regulatory Agency, and distribute the product throughout Brazil via the Unified Health System network.

## Conclusion

Peripheral nerves inherently possess the ability to heal after an injury. Fibrin sealants have proven to be valuable in regenerative and surgical applications, particularly in cases of nerve injury. However, there’s a limited recovery window before tissue degeneration obstructs the regeneration process and reconnection to the target. Despite the high costs and use of human blood associated with commercial sealants, HFB presents as a viable alternative. The use of HFB, in conjunction with conventional sutures, has been shown to enhance this regenerative effect, also reducing the number of suture points and surgical time. Moreover, HFB has an enhancing potential as a scaffold for other adjunct therapies, such as cell-based therapies and drug delivery systems, further improving the regenerative process. 

Heterologous fibrin biopolymer provides a more conducive regenerative environment for nerves, muscles, and associated NMJs compared to sutures alone and has several advantages in relation to other fibrin sealants. Indeed, it has demonstrated a good capability of maintaining a less degenerated NMJ microenvironment and promoting early onset regeneration after injury. While clinical trials are necessary to establish definitively the benefits of fibrin sealants in human nerve repair, HFB shows significant promise as a potent bioproduct ready for clinical trials and eventual integration into clinical settings. Further research is warranted to determine if its use over extended periods post-treatment could lead to improved functional outcomes. The CMDO initiative will facilitate the translation of biopharmaceuticals from bench to bedside and pave the way for the international expansion of HFB.

### Abbreviations

NMJ: neuromuscular junction; HFB: heterologous fibrin biopolymer; CNS: central nervous system; Sb: synaptic button; ACh: acetylcholine; CEVAP: Center for the Study of Venoms and Venomous Animals; CMDO: contract development and manufacturing organizations; nAChRs: nicotinic acetylcholine receptors; tSC: terminal Schwann cell; AChE: acetylcholinesterase; MMP3: matrix metalloproteinase 3; NCAMS: neural cell adhesion molecules; WD: Wallerian degeneration; DAMPs: damage-associated molecular patterns; TNF: tumor necrosis factor; IL-: interleukin; M1: pro-inflammatory type 1 macrophages; M2: anti-inflammatory type 2 macrophages; VEGF-A: vascular endothelial growth factor; MuSK: muscle-specific kinase; Dok7: docking protein 7; FDA: Food and Drug Administration; HIV: human immunodeficiency virus; LLLT: low-level laser therapy; PCL: polycaprolactone; PNS: peripheral nerve system; MC: mononuclear cells; MSCs: mesenchymal stem cells; IA: intramedullary axotomy; hESCs: human embryonic stem cells; DF: dimethyl fumarate; BBFN: buccal branch of the facial nerve; PBM: photobiomodulation.

## Data Availability

Not applicable.
